# An Anti-Range-Deception-Jamming Method for Networked Moving Radar Based on Trajectory Optimization

**DOI:** 10.3390/s25154675

**Published:** 2025-07-29

**Authors:** Xiaofei Han, Huafeng He, Chuan He, Qi Zhang, Liyuan Wang, Tao Zhou, Xin Zhang

**Affiliations:** Department of Control Engineering, Rocket Force University of Engineering, Xi’an 710025, China; hanxf0806@163.com (X.H.);

**Keywords:** networked moving radar, anti-false-target-jamming, FT misjudgment probability, trajectory optimization

## Abstract

Aiming at the problem that the anti-range-deception-jamming effect of a networked moving radar system is severely affected by the spatial distribution of each radar, an anti-range-deception-jamming method for networked moving radar based on trajectory optimization is proposed. Firstly, the anti-jamming method of networked moving radar considering the radar position error (RPE) is proposed. Then, the theoretical expression for the false target (FT) misjudgment probability of networked moving radar is deduced. Based on the theoretical expression, a trajectory optimization model is formulated to minimize FT misjudgment probability. Simulation experiments validate both the correctness of the derived probability expression and the significant influence of the radar spatial distribution position on the FT misjudgment probability. Moreover, the simulation results verify that the proposed anti-jamming method can effectively reduce the FT misjudgment probability of networked moving radar under the condition of a high discrimination probability of the physical target (PT).

## 1. Introduction

Modern electronic countermeasures (ECM) and electronic counter-countermeasures (ECCM) are continuously developing. Active range deception false target (FT) jamming is an important method in radar jamming technology [1,2]. The FT jamming is generated by the jammer modulating and transmitting the intercepted radar signal, thereby interfering with the target detection, positioning, and tracking in the radar system [3,4,5]. Monostatic radar has many mature approaches to counter deception jamming. For instance, optimized signals [6,7], motion feature [8], polarization information [9,10], digital radio frequency memory (DRFM) quantization errors [11,12], and frequency diversity [13,14] are used to discriminate FTs. However, ECM technology has advanced significantly with the rapid development of DRFM and electronic devices. Consequently, anti-jamming methods based on networked radar systems have been gradually proposed in recent years. The networked radar system consists of multiple spatially separated receiving stations, transmitting stations, or transceiver stations. Due to the spatial separation, the networked radar system can perceive the environment from multiple perspectives and dimensions. In contrast to the networked radar system, the monostatic radar can only detect the environment from a single perspective, resulting in less information and limited anti-jamming capabilities [15,16,17]. Moreover, the application of information fusion technology can effectively improve the anti-jamming performance of networked radar systems [18,19].

Anti-jamming methods for networked radar systems can be classified into two types: signal-level fusion methods and data-level fusion methods. The signal-level fusion method refers to cooperative processing where echo signals from each radar station are transmitted to a unified fusion center, and the amplitude and phase information of these signals is fused for anti-jamming processing [20]. For active deception jamming, current signal-level fusion methods mainly rely on the differences in spatial scattering characteristics to discriminate between physical targets (PTs) and false targets (FTs) [21]. In [22], an FT discrimination method based on the difference in spatial scattering characteristics was proposed, but it cannot guarantee a constant rejection ratio for PTs. Therefore, a new signal fusion method using the Neyman–Pearson lemma was proposed in [23]. Both refs. [22,23] discriminate FTs under the condition that the PT echo signals are completely independent in different receiving stations. However, in networked radar systems, the correlation of PT echoes in different directions depends on the target’s spatial position relative to the radar station. In different stations, PT echoes are not always spatially independent; more commonly, they are partially correlated. In the case of partly correlated PT echoes, a deception FT discrimination method was proposed in [24]. In the afore-mentioned references, echo signals with multiple pulse repetition intervals (PRIs) are used to estimate the correlation. To address this challenge, ref. [25] proposed a novel target discrimination method based on the difference in amplitude ratio feature. In [26], the hierarchical clustering analysis method was first applied in networked radar systems to discriminate deception targets within one PRI in a defined amplitude ratio feature space. The methods mentioned in references [22,23,24,25,26] can only be used for independent target detection at each radar station. When the networked radar system cooperatively detected targets, two signal-level anti-deception-jamming methods based on the likelihood-ratio test [27] and parameter estimation [28] were further proposed. Additionally, based on the differences between target echoes and deception jamming in scattering coefficient properties, a two-stage detection/discrimination algorithm was studied by applying the Neyman–Pearson (NP) criterion and a hybrid NP-Maximum Posteriori (MAP) criterion [29]. In addition, many methods for identifying FTs based on parameter estimation have been proposed [28,30]. In the afore-mentioned signal-level fusion anti-jamming methods, the amplitude and phase signals of the echoes in networked radar systems are used for fusion processing, which imposes high requirements for data transmission and signal time alignment. Therefore, the signal-level fusion anti-jamming method faces some difficulties in practical engineering applications.

In data-level anti-jamming methods, the angle, position, and velocity information of targets at each radar station are used to distinguish PTs from FTs [31]. In [32], based on the spatial consistency of PTs and the spatial dispersity of FTs, the homology hypothesis test method was proposed, which effectively discriminates FTs. However, the algorithm complexity increases when there are many FTs in interference scenarios. To address this problem, a method based on multiple discrimination for multi-range-false-target jamming was proposed in [31], which reduces the discrimination time. Based on the spatial distribution differences in position and velocity between PTs and FTs, the position and velocity information were used to identify FTs in [33]. Aiming at the distribution differences in target tracks between PTs and FTs, a phantom track jamming recognition method based on mean-variance collaborative testing was proposed in [34], which can effectively discriminate the tracks of FTs. The afore-mentioned references belong to data-level fusion anti-jamming methods based on centralized architectures. In [35], an anti-distance-deception-jamming method was proposed for active/passive networked radar systems with distributed fusion architectures. When the target is located far away from the radar or the angle between the target and active/passive radar is small, the anti-jamming effect of this method is negatively affected. After optimizing the above algorithm in [34], radial velocity information was added to identify PTs and FTs in the track initiation stage in [36], which effectively improves the FT discrimination effect of isomerism networked moving radar. In summary, most data-level fusion anti-jamming methods are currently applicable to ground-based networked radar systems. Only ref. [36] considers the radar position error (RPE) in isomerism networked radar systems. An isomerism radar networked system refers to a multisite radar system integrating different types of radar stations. An isomorphism radar networked system denotes a multisite system in which all radar stations are of the same type.

In summary, research on multistatic radar systems mostly focuses on ground platforms, and anti-jamming methods for networked moving radars are rarely reported. Since the networking system is constantly moving, the corresponding anti-jamming methods need to consider the radar position error (RPE). Moreover, the anti-jamming effect of networked radar systems is easily influenced by the spatial position of each radar. Therefore, in a deception jamming environment, trajectory optimization of networked moving radar is crucial for improving the effectiveness of FT discrimination. The proposed method aims to address FT discrimination challenges in radar networks deployed on moving platforms. This paper focuses on the anti-jamming method for isomorphism networked moving radar systems based on trajectory optimization. The method comprises two principal components: trajectory optimization and anti-jamming processing for networked moving radar systems. The main contributions of this paper are as follows:

(1) An anti-range-deception-jamming method for isomorphism networked moving radar is proposed, which considers the RPE and ensures a higher PT discrimination probability, i.e., $P_{T}$.
(2) The mathematical model of the FT misjudgment probability $P_{f}^{\prime }$ for isomorphism networked moving radar is derived.
(3) The trajectory optimization model for networked moving radar is established by minimizing the FT misjudgment probability as the objective function. Specifically, the motion constraints of networked radar are appropriately set. Additionally, the particle swarm optimization (PSO) algorithm is applied to solve the objective function, obtaining the optimal trajectory.

The rest of the paper is organized as follows. Section 2 introduces the anti-range-deception-jamming method for isomorphism networked moving radar. The mathematical model of the FT misjudgment probability is presented in Section 3. Section 4 constructs the trajectory optimization model for networked moving radar. In Section 5, the mathematical model of the FT misjudgment probability is verified by simulations. In particular, the PT/FT discrimination effect of the proposed anti-jamming method is demonstrated. Finally, conclusions are presented in Section 6.

## 2. Anti-Range-Deception-Jamming Method for Networked Moving Radar

**Figure 1:**
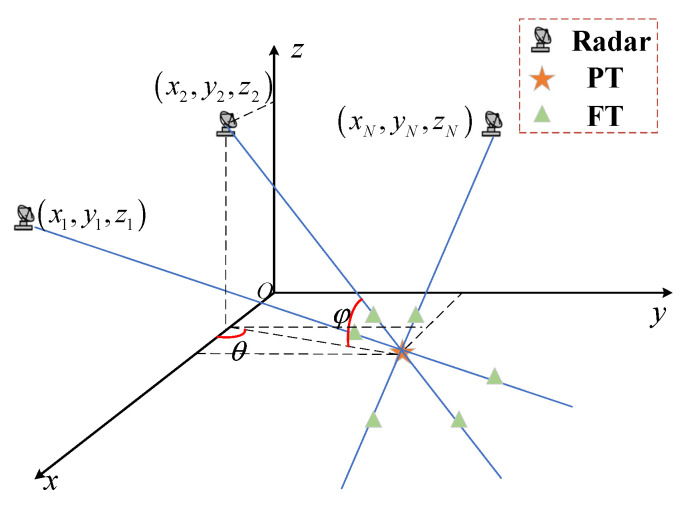
The scenario for countering range deception jamming in the networked moving radar.

Range deception jamming is discussed in this paper. This paper configures the simulation scenario with networked moving radars as airborne platforms and PTs positioned on ground or sea surfaces. The jammer and target are situated in the far-field region, and the jammer is positioned on or near the PT. Consequently, the jammer and PT coincide spatially. The range deception FTs generated by the jammer lie along the radial line connecting the PT and the radar. The scenario for countering range deception jamming in networked moving radar systems is illustrated in Figure 1.

Assuming that there are N radars in the networked radar system, two radars are taken as an example to illustrate the anti-range-deception-jamming method of networked moving radar. The positions of the two radars can be set as $\left(x_{1},y_{1},z_{1}\right)$, $\left(x_{2},y_{2},z_{2}\right)$, and the corresponding target measurements are $\left(r_{1},\theta_{1},\varphi_{1}\right)$, $\left(r_{2},\theta_{2},\varphi_{2}\right)$, respectively. Let $R_{i}=r_{i}\cos\varphi_{i}\left(i=1,2\right)$. The measurements of the two radars are converted into a unified rectangular coordinate system, and their coordinates in the xy-plane can be expressed as

(1) $$Z_{i}={\left[x_{T_{i}},y_{T_{i}}\right]}^{T},\left(i=1,2\right)$$

where

(2) $$\left\{\begin{array}{l} x_{T_{i}}=r_{i}\cos\varphi_{i}\cos\theta_{i}+x_{i}\\ y_{T_{i}}=r_{i}\cos\varphi_{i}\sin\theta_{i}+y_{i} \end{array}\right.$$

The measurement errors of each radar approximately obey a Gaussian distribution with zero-mean. And the error covariance matrix $P_{i}$ of $Z_{i}$ can be calculated by

(3) $$P_{i}=T_{i}\Lambda_{i}T_{i}^{T}$$

where $T_{i}$ represents the transformation matrix. $\Lambda_{i}$ is a diagonal matrix composed of the radar measurement errors and the RPE.

(4) $$T_{i}=\left[\begin{array}{c} \frac{\partial x_{T_{i}}}{\partial \left(r_{i}\cos\varphi_{i}\right)},\frac{\partial x_{T_{i}}}{\partial \theta_{i}},\frac{\partial x_{T_{i}}}{\partial x_{i}},\frac{\partial x_{T_{i}}}{\partial y_{i}}\\ \frac{\partial y_{T_{i}}}{\partial \left(r_{i}\cos\varphi_{i}\right)},\frac{\partial y_{T_{i}}}{\partial \theta_{i}},\frac{\partial y_{T_{i}}}{\partial x_{i}},\frac{\partial y_{T_{i}}}{\partial y_{i}} \end{array}\right]=\left[\begin{array}{c} \cos\theta_{i},-r_{i}\cos\varphi_{i}\sin\theta ,1,0\\ \sin\theta_{i},r_{i}\cos\varphi_{i}\cos\theta ,0,1 \end{array}\right]$$

(5) $$\begin{array}{cc} \Lambda_{i} & =\mathrm{diag}(\left[\frac{\partial (r_{i}\cos\varphi_{i})}{\partial r_{i}},\frac{\partial (r_{i}\cos\varphi_{i})}{\partial \varphi_{i}}\right][\begin{array}{cc} \sigma_{r_{i}}^{2} & 0\\ 0 & \sigma_{\varphi_{i}}^{2} \end{array}]{\left[\frac{\partial (r_{i}\cos\varphi_{i})}{\partial r_{i}},\frac{\partial (r_{i}\cos\varphi_{i})}{\partial \varphi_{i}}\right]}^{T},\sigma_{\theta_{i}}^{2},\sigma_{x_{i}}^{2},\sigma_{y_{i}}^{2})\\ & =[\begin{array}{cccc} \left[\cos\varphi_{i}-r\sin\varphi_{i}][\begin{array}{cc} \sigma_{r_{i}}^{2} & 0\\ 0 & \sigma_{\varphi_{i}}^{2} \end{array}][\begin{array}{c} \cos\varphi_{i}\\ -r\sin\varphi_{i} \end{array}\right] & & & \\ & \sigma_{\theta_{i}}^{2} & & \\ & & \sigma_{x_{i}}^{2} & \\ & & & \sigma_{y_{i}}^{2} \end{array}] \end{array}$$

where $\sigma_{r_{i}}$, $\sigma_{\theta_{i}}$, and $\sigma_{\varphi_{i}}$ represent the standard deviations of the range measurement, azimuth angle measurement, and elevation angle measurement in the *i*-th radar, respectively.$\sigma_{x_{i}}$ and $\sigma_{y_{i}}$ are the standard deviations of the radar position errors in the x-axis and y-axis, separately.

Let $\Delta Z=Z_{1}-Z_{2}$. If the measurements of the two radars correspond to the same PT, ΔZ obeys a Gaussian distribution with zero-mean. Since the measurement errors of the two radars are independent of each other, the error covariance matrix $\Sigma_{12}$ of ΔZ can be expressed as

(6) $$\Sigma_{12}=E\left[\left(dZ_{1}-dZ_{2}\right){\left(dZ_{1}-dZ_{2}\right)}^{T}\right]=P_{1}+P_{2}$$

The Mahalanobis distance can be obtained by

(7) $$d^{2}=\Delta Z^{T}\Sigma_{12}^{-1}\Delta Z$$

Let $H_{0}$ represent the measurements of the two radars corresponding to the same PT. Let $H_{1}$ represent the measurements of the two radars corresponding to different targets. The hypothesis testing model can be expressed as

(8) $$\left\{\begin{array}{c} H_{0}:d^{2}\leq \delta \\ H_{1}:d^{2}>\delta \end{array}\right.$$

where $\delta =\chi_{\alpha }^{2}(2)$ is the threshold value, which can be acquired by querying the chi-square distribution table. Under hypothesis $H_{0}$, the Mahalanobis distance $d^{2}$ approximately follows the chi-square distribution with the significance level of α and the freedom degree of 2. α represents the probability that the hypothesis test result is wrong. In order to make the hypothesis test results more accurate, the Mahalanobis distances at multiple moments are accumulated as a test statistic, which can be described as

(9) $$\Gamma =\sum_{k=1}^{K}d_{k}^{2}\left(k=1,2,\cdots ,K\right)$$

where *K* is the accumulation time of the test statistic, and $d_{k}^{2}$ is the Mahalanobis distance at the *k*-th moment.

If $\Gamma \leq \chi_{\alpha }^{2}\left(2K\right)$ this means that the measurements of the two radars correspond to the same target (i.e., the PT). Otherwise, $\Gamma >\chi_{\alpha }^{2}\left(2K\right)$ means that the targets detected by the two radars correspond to different targets. If the number of radars is more than 2 (i.e., N>2), the system combines radars in pairs to discriminate FTs. To maximize the PT discrimination probability, the system labels a target as an FT only if all pairwise combinations classify it as an FT.

## 3. Mathematical Model of FT Misjudgment Probability

Note that the FT misjudgment probability refers to the probability of an FT being erroneously identified as a PT. The trajectory optimization model for the networked radar system aims to minimize the FT misjudgment probability as its objective function. To achieve this, the mathematical model of the FT misjudgment probability must be rigorously derived. A two-radar scenario is first examined as a foundational case.

Measurements of FTs from both radars are transformed into a unified rectangular coordinate system. The corresponding position coordinates in the xy-plane are expressed as

(10) $$Z_{i}=\left(\begin{array}{c} x_{F_{i}}\\ y_{F_{i}} \end{array}\right)=\left(\begin{array}{c} \left(r_{i}\cos\varphi_{i}+\Delta r\right)\cos\theta_{i}+x_{i}\\ \left(r_{i}\cos\varphi_{i}+\Delta r\right)\sin\theta_{i}+y_{i} \end{array}\right),\left(i=1,2\right)$$

where Δr denotes the deception range. If the coordinates correspond to a PT, the coordinate difference ΔZ follows a zero-mean normal distribution with covariance matrix $\Sigma_{12}$. If the coordinates correspond to FTs, ΔZ follows a normal distribution with mean E and covariance matrix $\Sigma_{12}^{\prime }$, which can be described as

(11) $$\left\{\begin{array}{c} H_{0}:\Delta Z\sim N\left(0,\Sigma_{12}\right)\\ H_{1}:\Delta Z\sim N\left(E,\Sigma_{12}^{\prime }\right) \end{array}\right.$$

where

(12) $$E={\left[\mu_{x},\mu_{y}\right]}^{T}={\left(x_{F_{1}},y_{F_{1}}\right)}^{T}-{\left(x_{F_{2}},y_{F_{2}}\right)}^{T}$$

The calculation matrix $\Sigma_{12}^{\prime }$ is computed analogously to $\Sigma_{12}$ (details omitted for brevity). As a symmetric matrix, $\Sigma_{12}^{\prime }$ represents the measurement error covariance between the two radars and can be rewritten as

(13) $$\Sigma_{12}^{\prime }=\left[\begin{array}{cc} \xi_{11} & \xi_{12}\\ \xi_{21} & \xi_{22} \end{array}\right]=\left[\begin{array}{cc} \sigma_{x}^{2} & \rho \sigma_{x}\sigma_{y}\\ \rho \sigma_{x}\sigma_{y} & \sigma_{y}^{2} \end{array}\right]$$

where $\xi_{12}=\xi_{21}$, $\sigma_{x}^{2}$, and $\sigma_{y}^{2}$ denote the measurement error variances along the x-axis and y-axis, respectively, while $\rho =\xi_{12}/\sigma_{x}\sigma_{y}$ represents the correlation coefficient between these errors. The two-dimensional probability density function (PDF) of ΔZ is given by

(14) $$f\left(x,y\right)=\frac{1}{2\pi \sigma_{x}\sigma_{y}\sqrt{1-\rho^{2}}}\exp\left\{\frac{-1}{2\left(1-\rho^{2}\right)}\times \left[\frac{{\left(x-\mu_{x}\right)}^{2}}{\sigma_{x}^{2}}-2\rho \frac{\left(x-\mu_{x}\right)\left(y-\mu_{y}\right)}{\sigma_{x}\sigma_{y}}+\frac{{\left(y-\mu_{y}\right)}^{2}}{\sigma_{y}^{2}}\right]\right\}$$

The Mahalanobis distance $d^{2}=\Delta Z^{T}\Sigma_{12}^{\prime -1}\Delta Z$ simplifies as

(15) $$\begin{array}{cc} d^{2} & =\Delta Z^{T}\Sigma_{12}^{\prime -1}\Delta Z\\ & ={\left[Z_{1}-Z_{2}\right]}^{T}\Sigma_{12}^{\prime -1}\left[Z_{1}-Z_{2}\right]\\ & ={\left[\begin{array}{c} x_{F_{1}}-x_{F_{2}}\\ y_{F_{i}}-y_{F_{2}} \end{array}\right]}^{T}\Sigma_{12}^{\prime -1}\left[\begin{array}{c} x_{F_{1}}-x_{F_{2}}\\ y_{F_{i}}-y_{F_{2}} \end{array}\right]\\ & ={\left[\begin{array}{c} x\\ y \end{array}\right]}^{T}{\left[\begin{array}{cc} \sigma_{x}^{2} & \rho \sigma_{x}\sigma_{y}\\ \rho \sigma_{x}\sigma_{y} & \sigma_{y}^{2} \end{array}\right]}^{-1}[\begin{array}{c} x\\ y \end{array}]\\ & =\frac{1}{\sigma_{x}^{2}\sigma_{y}^{2}\left(1-\rho^{2}\right)}{\left[\begin{array}{c} x\\ y \end{array}\right]}^{T}[\begin{array}{cc} \sigma_{y}^{2} & -\rho \sigma_{x}\sigma_{y}\\ -\rho \sigma_{x}\sigma_{y} & \sigma_{x}^{2} \end{array}][\begin{array}{c} x\\ y \end{array}]\\ & =\frac{1}{\sigma_{x}^{2}\sigma_{y}^{2}\left(1-\rho^{2}\right)}(x^{2}\sigma_{y}^{2}-2xy\rho \sigma_{x}\sigma_{y}+y^{2}\sigma_{x}^{2})\\ & =\frac{1}{\left(1-\rho^{2}\right)}[{\left(\frac{x}{\sigma_{x}}\right)}^{2}-2\rho (\frac{x}{\sigma_{x}})(\frac{y}{\sigma_{y}})+{\left(\frac{y}{\sigma_{y}}\right)}^{2}] \end{array}$$

From this analysis, the FT misjudgment probability for the two-radar system is derived as

(16) $$\begin{array}{cc} P_{12} & =P\left(H_{0}|H_{1}\right)\\ & =P\left(d^{2}\leq \delta |H_{1}\right)\\ & =P(\frac{1}{\left(1-\rho^{2}\right)}\left[{\left(\frac{x}{\sigma_{x}}\right)}^{2}-2\rho \left(\frac{x}{\sigma_{x}}\right)\left(\frac{y}{\sigma_{y}}\right)+{\left(\frac{y}{\sigma_{y}}\right)}^{2}\right]\leq \delta |H_{1}) \end{array}$$

By defining

(17) $$\Omega_{12}=\left\{\frac{1}{\left(1-\rho^{2}\right)}\left[{\left(\frac{x}{\sigma_{x}}\right)}^{2}-2\rho \left(\frac{x}{\sigma_{x}}\right)\left(\frac{y}{\sigma_{y}}\right)+{\left(\frac{y}{\sigma_{y}}\right)}^{2}\right]\leq \delta \right\}$$

the FT misjudgment probability becomes a double integral of the PDF $f\left(x,y\right)$ over the region $\Omega_{12}$, i.e.,

(18) $$P_{12}=\iint_{\Omega_{12}}f\left(x,y\right)dxdy$$

Letting

(19) $$X=\frac{x}{\sigma_{x}},Y=\frac{y}{\sigma_{y}},A=\left(1-\rho^{2}\right)\delta$$

Equation (17) can be simplified as

(20) $$\begin{array}{cc} \Omega_{12} & =\left\{X^{2}-2\rho XY+Y^{2}\leq A\right\}\\ & =\left\{X^{2}-2\rho XY+\rho^{2}Y^{2}+\left(1-\rho^{2}\right)Y^{2}\leq A\right\}\\ & =\left\{{\left(X-\rho Y\right)}^{2}\leq A-\left(1-\rho^{2}\right)Y^{2}\right\} \end{array}$$

Finally, the FT misjudgment probability $P_{12}$ is computed via

(21) $$P_{12}=\iint_{\Omega_{12}}f\left(x,y\right)dxdy=\int_{-\sigma_{y}\sqrt{\left(1-\rho^{2}\right)\delta }}^{\sigma_{y}\sqrt{\left(1-\rho^{2}\right)\delta }}\int_{l_{low}\left(y\right)}^{l_{up}\left(y\right)}f\left(x,y\right)dxdy$$

(22) $$l_{low}\left(y\right)=\left(\rho \frac{y}{\sigma_{y}}-\sqrt{\left(1-\rho^{2}\right)\left[\delta -{\left(\frac{y}{\sigma_{y}}\right)}^{2}\right]}\right)\sigma_{x}$$

(23) $$l_{up}\left(y\right)=\left(\rho \frac{y}{\sigma_{y}}+\sqrt{\left(1-\rho^{2}\right)\left[\delta -{\left(\frac{y}{\sigma_{y}}\right)}^{2}\right]}\right)\sigma_{x}$$

In a networked radar system, FTs are identified by pairwise radar combinations. A target is classified as an FT only if all the pairwise identification results concur. Consequently, the FT misjudgment probability model $P_{f}^{\prime }$ for the networked moving radar system generalizes the two-radar case as

(24) $$P_{f}^{\prime }=\prod_{i,j=1,2,\cdots ,N;i\neq j}P_{ij}$$

where N is the number of radars in the networking system. $P_{ij}$ represents the FT misjudgment probability for the pairwise combination of the *i*-th and the *j*-th radars, which can be obtained by

(25) $$P_{ij}=\iint_{\Omega_{ij}}f_{ij}\left(x,y\right)dxdy$$

## 4. Trajectory Optimization Method for Networked Moving Radar

The proposed trajectory optimization model for networked moving radars, designed for two-dimensional scenarios, aims to minimize the FT misjudgment probability. This optimization problem can be decomposed into multistage deployment subproblems with distinct constraints at different time instances. The constraints considered are as follows:

(1) Detection Range Constraint: The target area must always lie within the maximum detection range of the radars.
(2) Multi-Perspective and Interference Constraint: To ensure multi-perspective advantages and avoid signal interference, a minimum safety distance between any two radars must be maintained.
(3) Flight Direction Constraint: All radars must fly toward the target area.

Notably, the constraints at the initial time instance differ from those at subsequent stages. The constraints for the initial and subsequent phases should be described separately. Specifically, the initial phase considers radar deployment boundaries and starting positions, while subsequent phases incorporate dynamic mobility limits such as positional/angular variation bounds. The initial phase is defined as the period when the proposed anti-jamming method is first applied by the networked moving radar.

Let the target area D be centered at position vector $X_{T}$. For the *i*-th radar at the *k*-th moment, denote its position vector as is $X_{i}^{k}$, $\left(i=1,\cdots ,N;k=1,\cdots ,K\right)$. The objective function of minimizing the FT misjudgment probability is formulated as

(26) $$fit_{best}=\underset{X_{i}^{k}\left(i=1,\cdots ,N;k=1,\cdots ,K\right)}{\min}\sum_{D}\prod_{i,j=1,2,\cdots ,N;i\neq j}P_{ij}$$

where $fit_{best}$ is the optimal fitness value for the trajectory optimization algorithm.

**Figure 2:**
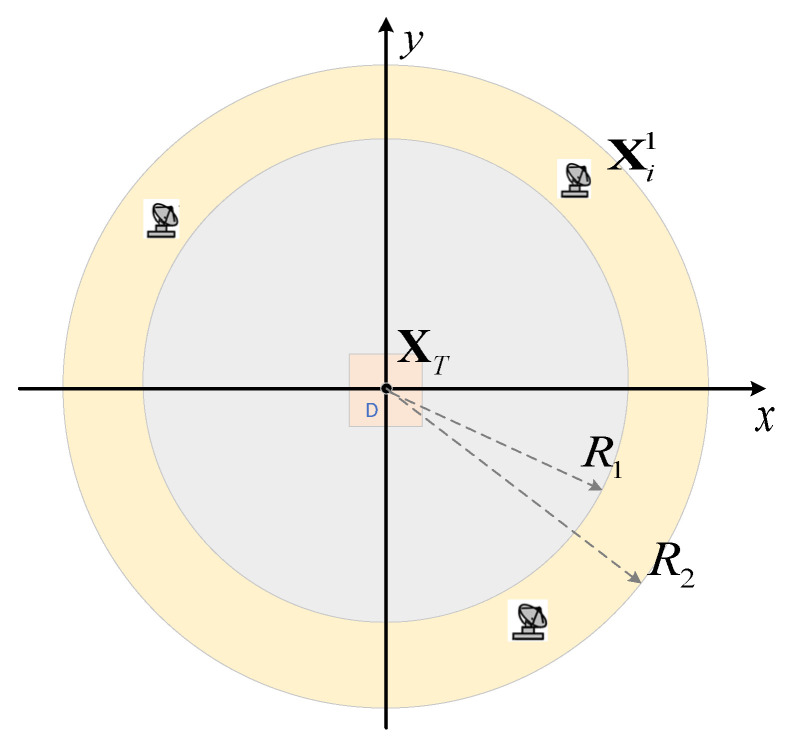
Optimization range at the first moment.

The constraints at the initial phase can be described as

(27) $$\begin{array}{l} R_{1}\leq \left\|X_{i}^{1}-X_{T}\right\|\leq R_{2}\\ \left\|X_{i}^{1}-X_{j}^{1}\right\|\geq \Delta R_{\min} \end{array}\left(i,j=1,2,\cdots ,N;i\neq j\right)$$

where $R_{1}$ and $R_{2}$ determine the optimization range of networked radar at the first moment and ensure that the target area is within the radar detection range. $\Delta R_{\min}$ represents the minimum distance between two radars. At the first moment, the optimization range is shown in the yellow circle in Figure 2.

The constraints at subsequent phases can be described as

(28) $$\begin{array}{ll} \left\|X_{i}^{1}-X_{j}^{1}\right\|\geq \Delta R_{\min}\\ r_{best}^{k-1}-\Delta r_{\max}\leq R_{X_{i}}^{k}\leq r_{best}^{k-1}-\Delta r_{\min} & \left(i,j=1,2,\cdots ,N;i\neq j\right)\\ \theta_{best}^{k-1}-\Delta \theta \leq \theta_{X_{i}}^{k}\leq \theta_{best}^{k-1}+\Delta \theta \end{array}$$

where

- $R_{X_{i}}^{k}$ represents the distance between the radar and the target area center $X_{T}$ at the *k*-th moment.
- $r_{best}^{k-1}$ is the distance between the radar optimal position $X_{best}^{k-1}$ and the target area center $X_{T}$ at the $\left(k-1\right)\mathrm{th}$ moment.
- $\Delta r_{\max}$ represents the maximum variation of $r_{best}^{k-1}$.
- $\Delta r_{\min}$ represents the minimum variation of $r_{best}^{k-1}$.
- $\theta_{X_{i}}^{k}$ represents the radar azimuth angle at the *k*-th moment.
- $\theta_{best}^{k-1}$ is the azimuth angle of the radar optimal position at the $\left(k-1\right)-\mathrm{th}$ moment.
- Δθ indicates the maximum angle variation.

**Figure 3:**
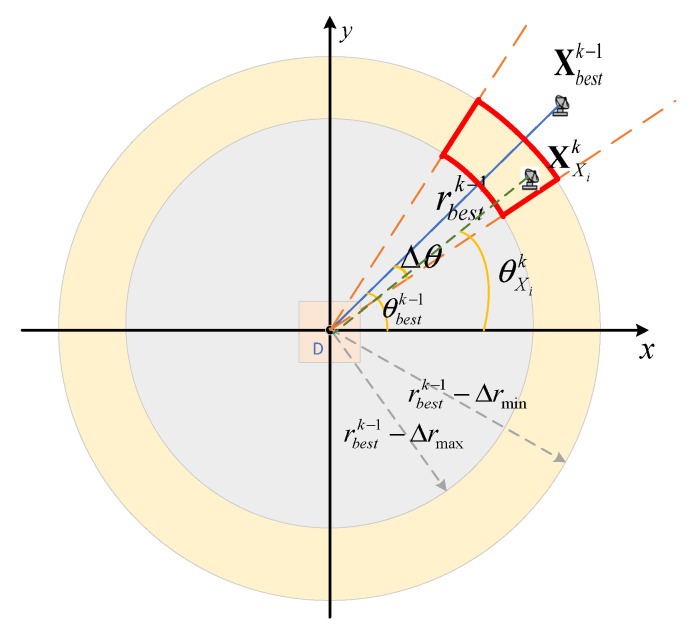
Constraint condition at the following moments.

These constraints are illustrated in Figure 3. The area enclosed by the red curve represents the optimization area of the radar at the next moment.

The optimal trajectory of the networked moving radar is determined by the optimized positions at multiple time instances. The hypothesis test statistic value is computed by calculating the Mahalanobis distance at each moment and then summing them. Finally, the FT can be discriminated by the anti-jamming method of networked moving radar. The anti-FT-jamming process for networked moving radars based on trajectory optimization is shown in Table 1.

## 5. Numerical Simulations

### 5.1. Mathematical Model Validation of FT Misjudgment Probability

Taking two radars as an example, the mathematical model of the FT misjudgment probability is verified by the simulations. The positions of the two radars, along with the radar range measurement error (RME) and the angle measurement error (AME), are presented in Table 2. The deception range is set as 1000 m. The target areas in the x-axis and y-axis are $\left(-100\sim 150\right)\mathrm{km}$ and $\left(-50\sim 100\right)\mathrm{km}$, respectively. The coordinate value of the target area in the z-axis is 0km. The anti-range-deception-jamming method for the networked moving radar system is simulated 500 times based on the Monte Carlo method, and the statistical values of $P_{T}$ and $P_{f}^{\prime }$ can be obtained. These statistical results are compared with the theoretical results. It is worth noting that $P_{T}$ indicates the probability that the PT is judged correctly, and the ${P^{\prime }}_{f}$ represents the probability that an FT is mistaken for a PT.

**Figure 4:**
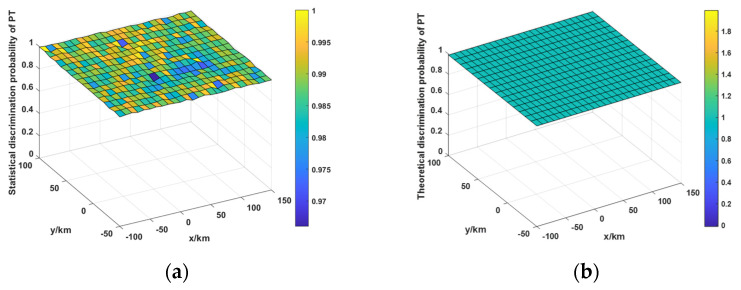
Discrimination probability of PT. (**a**) Statistical values of $P_{T}$, (**b**) Theoretical values of $P_{T}$.

**Figure 5:**
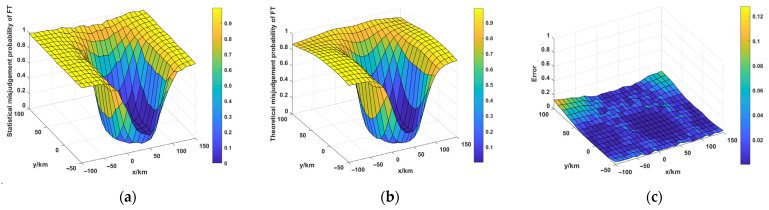
FT misjudgment probability of FT. (**a**) Statistical misjudgment probability of FT, (**b**) Theoretical misjudgment probability of FT, (**c**) Errors.

It can be seen from Figure 4 and Figure 5 that the statistical values of $P_{T}$ are relatively close to the theoretical values. And the errors between the statistical and theoretical results of $P_{f}^{\prime }$ are also relatively small. The simulation results validate the accuracy of the mathematical model for FT misjudgment probability.

### 5.2. Simulation Experiment of Trajectory Optimization

Assume that the networked moving radar system is composed of three radars. The PSO algorithm is utilized to solve the optimal trajectory at five moments. The target area D is set as a square area, and its coordinate ranges in the x-axis, y-axis, and z-axis are c, $\left(-2\times 10^{3}\sim 2\times 10^{3}\right)m$, and 0 m, respectively. The target area center is at the coordinate origin. Nine points are chosen uniformly to represent the entire target area, which are used to calculate the fitness value of the objective function. The deception range is set as 300 m. The RMEs of the three radars are all set to be 5 m, the AMEs are all 0.2°, and the RPEs in the x-axis, y-axis, and z-axis are all 100 m. It is assumed that the coordinates of the three radars in the z-axis are all 30 km.

The constraints at the first moment are different from those of the following four moments. The optimization time at the first moment is not restricted. Hence, when the optimal radar deployment is solved at the first moment, the population number and iteration number of the PSO algorithm can be set as relatively large. In the following four moments, the optimal position of networked radar needs to be solved during the movement process. The algorithm optimization speed must meet the real-time requirements. The number of populations and iterations at the following four moments need to be set as relatively small.

#### 5.2.1. Scenario 1

**Figure 6:**
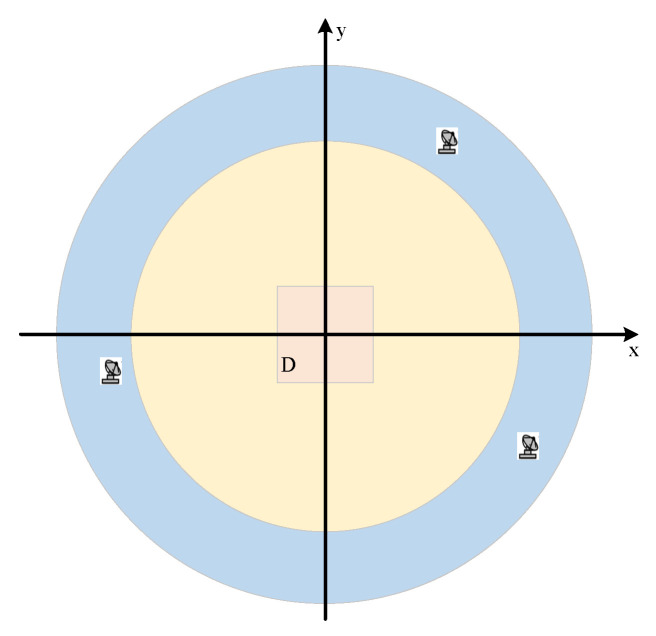
Trajectory optimization range in Scenario 1.

The center point of the trajectory optimization area is placed at the coordinate origin, and three radars are deployed moving toward the target area D from surrounding directions, as shown in Figure 6. The blue area represents the optimal deployment range of the networked radar system at the first moment. The yellow area represents the optimal deployment range of the networked system at the following four moments. The simulation parameters at the first moment are shown in Table 3, and the simulation parameters of the following four moments are shown in Table 4.

**Figure 7:**
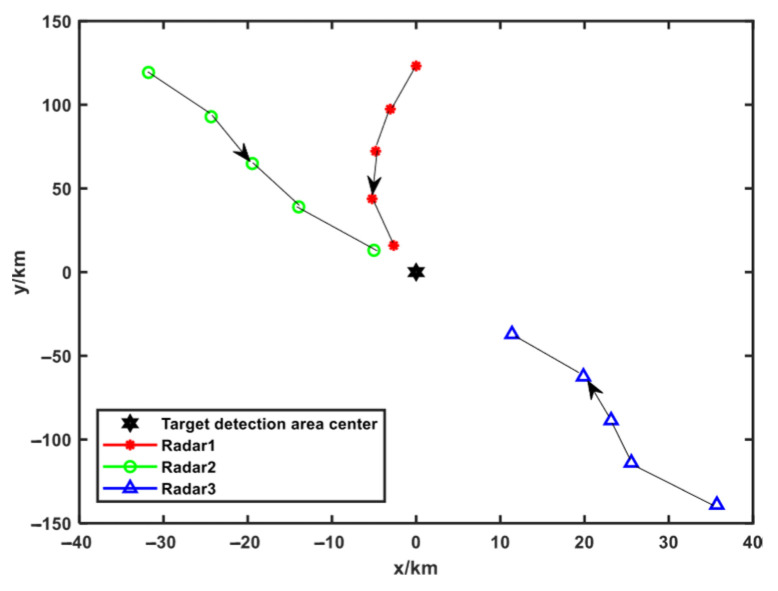
Trajectory optimization results of Scenario 1.

The trajectory optimization results are shown in Figure 7. The optimal objective function fitness value at each moment and the corresponding radar positions are given in Table 5. As shown in Figure 7, the positions of the three radars are relatively scattered to ensure the multi-perspective advantage of networked radar. Since nine points are selected to represent the entire target area, the maximum fitness value of the objective function is equal to 9.

#### 5.2.2. Scenario 2

**Figure 8:**
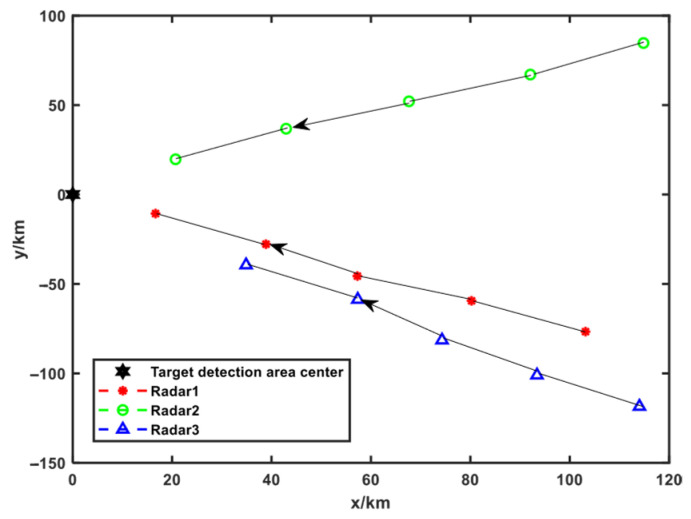
Trajectory optimization results of Scenario 2.

The center point of the trajectory optimization area is far away from the center point of the target area, which remains positioned at the coordinate origin. Since the networked radar system approaches target detection area D exclusively from the right-hand side, the trajectory optimization is constrained to the target area’s right sector. The trajectory optimization results in Scenario 2 are solved according to the trajectory optimization idea in Scenario 1, which are shown in Figure 8. The optimal objective function fitness value at each moment and the corresponding radar position coordinates are shown in Table 6.

### 5.3. Simulation Experiment of PT/FT Discrimination Effect

The simulation results of Scenario 2 are used as the moving trajectory of networked radar. The proposed anti-jamming method is compared with the existing anti-jamming method in [37], which discriminates FTs based on the position information and does not consider the RPE. The influence of the RPE, deception range, RME, and AME on the PT/FT discrimination effects is analyzed. It should be noted that the FT discrimination probability $P_{F}$ indicates the probability that the FT is judged correctly, i.e., $P_{F}=1-P_{f}^{\prime }$.

#### 5.3.1. Influence of RPE on Discrimination Probability

**Figure 9:**
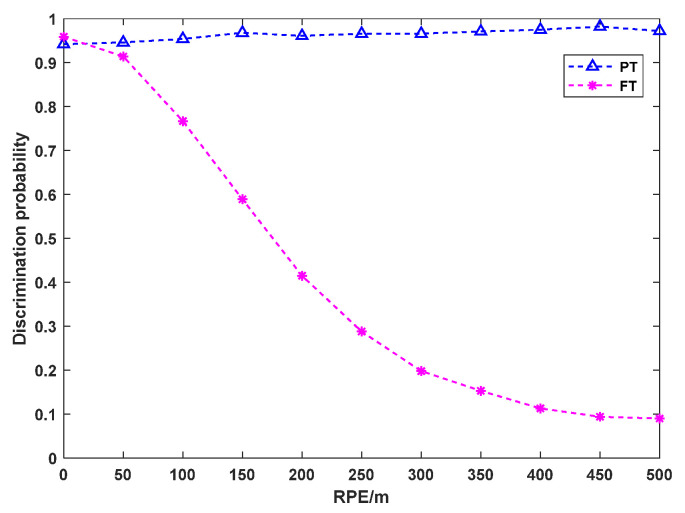
Influence of RPE on PT/FT discrimination probability.

The RMEs of the three radars are all 5 m, and the azimuth and elevation AMEs are both 0.2°. The deception range is set to 500 m. The number of Monte Carlo simulations is set to 1000. In the proposed method, the influence of the RPE on the PT/FT discrimination probability is shown in Figure 9.

As shown in Figure 9, with an increase in the RPE, the $P_{T}$ of the proposed method always remains at a high level, while the $P_{F}$ gradually decreases. Therefore, the radar with a smaller RPE can be selected for networking, which can effectively improve the $P_{F}$ of the networked system.

#### 5.3.2. Influence of Deception Range on Discrimination Probability

**Figure 10:**
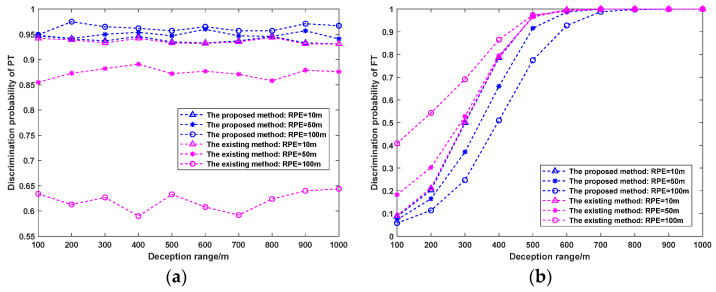
Influence of different deception ranges on PT/FT discrimination probability. (**a**) $P_{T}$, (**b**) $P_{F}$.

The radar parameters are not changed, and the number of Monte Carlo simulations is 1000. When the RPE is set to 10 m, 50 m, and 100 m, respectively, the influence of different deception ranges on the PT/FT discrimination probability is analyzed. The simulation results are given in Figure 10.

It can be seen from Figure 10a that with an increase in the deception range, the $P_{T}$ of the two methods shows little change. Nevertheless, with the rise of the RPE, the $P_{T}$ of the proposed method is always maintained at a high level, while the $P_{T}$ of the existing method is significantly reduced. Because the RPE is not considered in the existing method, the parameter values of the error covariance matrix $P_{i}\left(i=1,2,\cdots ,N\right)$ are incorrectly reduced. This leads to an inverse increase in the Mahalanobis distance. The test threshold is constant so that the PT is misjudged as an FT by the hypothesis test. Therefore, the $P_{T}$ of the existing method gradually decreases with the rise of the RPE.

As shown in Figure 10b, when the deception range increases, the $P_{F}$ of the two methods increases progressively. The $P_{F}$ of the proposed method progressively reduces with the increase in the RPE, while the $P_{F}$ of the existing method incrementally enhances. This is due to the effect of the RPE on the test statistic, which in turn affects the results of the hypothesis test. It is worth noting that it only makes sense to identify FTs when the PTs are not eliminated. Although the $P_{F}$ of the existing method is better than that of the proposed method, it is too costly in the $P_{T}$ of the existing method.

#### 5.3.3. Influence of RME on Discrimination Probability

**Figure 11:**
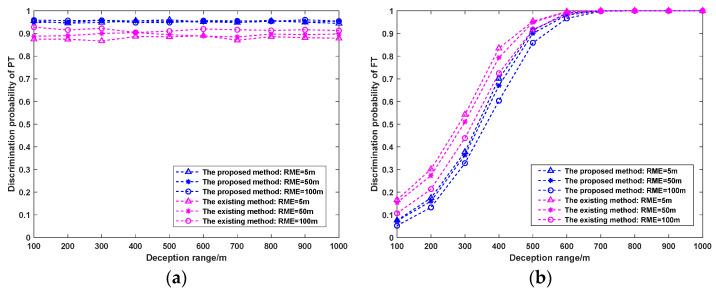
Influence of RME on PT/FT discrimination probability. (**a**) $P_{T}$, (**b**) $P_{F}$.

The RPEs of the three radars are all set to 50 m, and the measurement errors of the azimuth and elevation angles are all 0.2°. The number of Monte Carlo simulations is 1000, and the deception range is constantly changing. When the RME of the radar is 5 m, 50 m, or 100 m, the PT/FT discrimination effects of the two methods are analyzed. The simulation results are shown in Figure 11.

As can be seen from Figure 11, as the RME increases, the $P_{T}$ of both methods remains stable. The $P_{T}$ of the proposed method, which accounts for the RPE, is consistently higher than that of the existing method, whereas the $P_{F}$ is marginally lower. Additionally, both methods exhibit a slight decline in $P_{F}$ with increasing RME. These results indicate that improving the RME enhances the FT discrimination capability of the networked radar system.

#### 5.3.4. Influence of AME on Discrimination Probability

**Figure 12:**
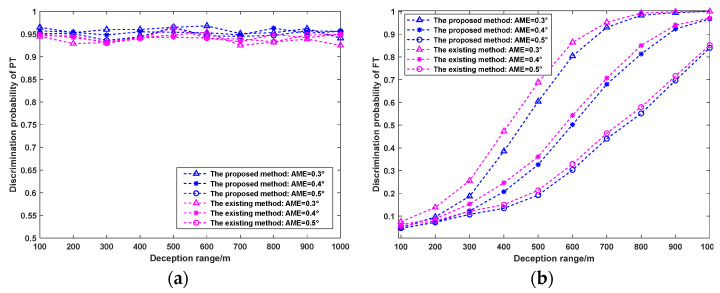
Influence of AME on PT/FT discrimination probability. (**a**) $P_{T}$, (**b**) $P_{F}$.

The RPEs of the three radars are all set to 50 m, and the RMEs are all 5 m. The Monte Carlo simulation is performed 1000 times, and the deception range is constantly changing. When the AMEs of the three radars are all 0.3°, 0.4°, and 0.5°, respectively, the PT/FT discrimination results of the two methods are shown in Figure 12.

As shown in Figure 12, the $P_{T}$ of the two methods is less affected by the AME, whereas the $P_{F}$ decreases gradually with increasing AME. Notably, $P_{F}$ exhibits higher sensitivity to AME compared to $P_{T}$. Selecting radars with smaller AMEs for networking can significantly enhance the FT discrimination performance of the radar network.

The influence of the parameters, including the RPE, deception range, RME, and AME, on $P_{T}$ and $P_{F}$ is summarized in Table 7. ↑ represents an increase, ↓ represents a decrease. As shown in Table 7, the deception range, RME, and AME exhibit consistent influence trends on the $P_{T}$ and $P_{F}$ for both methods. Only the RPE demonstrates divergent effects on $P_{T}$ and $P_{F}$ between the two methods. Analysis reveals that as the RPE increases, the existing method outperforms the proposed method in FT discrimination efficacy. However, this comes at the cost of a sharp decline in $P_{T}$ for the existing method. The proposed method always ensures a high PT. Critically, effective FT discrimination is meaningful only when PTs are accurately identified.

## 6. Conclusions

The existing anti-jamming methods for isomorphism networked radar systems are predominantly designed for ground-based radars. To address this limitation, this paper proposes an anti-jamming method for isomorphism networked moving radar that incorporates the RPE of each radar station. Given that radar positioning critically impacts the system’s anti-jamming performance, a trajectory optimization model is introduced. Firstly, the mathematical model for FT misjudgment probability in networked moving radar is first derived and analyzed. Subsequently, the trajectory optimization model is formulated. Through numerical simulations, the optimal trajectory minimizing FT misjudgment probability is obtained. The results demonstrate that the proposed anti-jamming method achieves effective FT identification while maintaining high PT detection accuracy. Although the proposed trajectory optimization method effectively minimizes FT misjudgment probability, future research could explore multi-objective trajectory optimization problems, such as simultaneously minimizing FT misjudgment probability and maximizing the detection coverage of the networked system. Additionally, by extending the FT misjudgment probability model and trajectory optimization model to three-dimensional scenarios, the core methodology can be generalized to three-dimensional scenarios. Future work will investigate trajectory-optimized countermeasures against range deception jamming for networked moving radars in three-dimensional scenarios.

## Figures and Tables

**Table 1:** Anti-jamming process for networked moving radars based on trajectory optimization.

**Input:** Coordinates of the target area D; Standard deviations of the distance measurement, the azimuth angle measurement, and the elevation angle measurement in the *i*-th radar $\sigma_{r_{i}}$, $\sigma_{\theta_{i}}$, $\sigma_{\varphi_{i}}$; Accumulation time of the test statistic *K*; Significance level α; Number of radars N.
**Output:** False target discrimination results.
1: Trajectory optimization: The optimal trajectory for discriminating FTs in the networked moving radar is determined using Equations (26)–(28).
2: PT/FT discrimination: Based on the optimal trajectory, the networked moving radar approaches the target area, with PTs and FTs being distinguished through the application of Equations (1)–(9).

**Table 2:** Radar parameters.

	Position	RME	Azimuth AME	Elevation AME	RPE
Radar 1	$\left(0,0,30\right)\mathrm{km}$	5 m	0.2°	0.2°	$\left(100,100,100\right)m$
Radar 2	$\left(80,0,30\right)\mathrm{km}$	5 m	0.2°	0.2°	$\left(100,100,100\right)m$

**Table 3:** Simulation parameters at the first moment of Scenario 1.

Time	Population Number	Iteration Number	$\left(R_{1}\sim R_{2}\right)$	Angle Change Range	$\Delta R_{\min}$
1	15	10	$\left(120\sim 150\right)\mathrm{km}$	0°~360°	10km

**Table 4:** Simulation parameters at the following four moments of Scenario 1.

Time	Population Number	Iteration Number	$\Delta r_{\max}$	$\Delta r_{\min}$	Δθ	$\Delta R_{\min}$
2	10	3	30km	25km	2°	10km
3	10	3	30km	25km	2°	10km
4	10	3	30km	25km	3°	10km
5	10	3	30km	25km	3°	10km

**Table 6:** Optimized radar position and optimal fitness value in Scenario 2.

Time	Radar 1		Radar 2		Radar 3		$\mathrm{Optimal}\;\mathrm{Fitness}\;\mathrm{Value}\;fit_{best}$
	x-Axis/km	y-Axis/km	x-Axis/km	y-Axis/km	x-Axis/km	y-Axis/km	
1	103.1696	−76.6429	114.8335	84.70966	114.0189	−118.504	7.2554
2	80.23781	−59.4082	92.07014	67.06041	93.40683	−100.99	7.2619
3	57.27299	−45.5741	67.65801	52.10995	74.29799	−81.4132	6.6083
4	38.88664	−27.7483	42.93435	36.80352	57.34919	−58.6715	5.2162
5	16.66467	−10.6208	20.69791	19.71265	34.88554	−39.415	3.6625

**Table 7:** The influence of parameters on $P_{T}$ and $P_{F}$.

Parameters	Proposed Method		Existing Method	
	$P_{T}$	$P_{F}$	$P_{T}$	$P_{F}$
RPE ↑	Basically unchanged	↓	↓	↑
Deception range ↑	Basically unchanged	↑	Basically unchanged	↑
RME ↑	Basically unchanged	↓	Basically unchanged	↓
AME ↑	Basically unchanged	↓	Basically unchanged	↓

**Table 5:** Optimized radar position and optimal fitness value in Scenario 1.

Time	Radar 1		Radar 2		Radar 3		$\mathrm{Optimal}\;\mathrm{Fitness}\;\mathrm{Value}\;fit_{best}$
	x-Axis/km	y-Axis/km	x-Axis/km	y-Axis/km	x-Axis/km	y-Axis/km	
1	0.00	123.19	−31.78	119.29	35.71	−139.06	2.1220
2	−3.07	97.40	−24.35	92.84	25.56	−113.92	2.1297
3	−4.80	72.25	−19.46	64.87	23.15	−88.61	2.2948
4	−5.23	43.83	−13.95	38.97	19.87	−62.46	2.7735
5	−2.66	15.91	−5.01	13.04	11.38	−37.12	3.9355

## Data Availability

The data presented in this study are available on request from the corresponding author.
